# Robust and rigorous identification of tissue-specific genes by statistically extending tau score

**DOI:** 10.1186/s13040-022-00315-9

**Published:** 2022-12-09

**Authors:** Hatice Büşra Lüleci, Alper Yılmaz

**Affiliations:** 1 Department of Bioengineering, Gebze Technical University, Kocaeli, Turkey; 2 Department of Bioengineering, Yildiz Technical University, Istanbul, Turkey

**Keywords:** Tissue-specific genes, RNA-Seq, Tau score

## Abstract

**Objectives:**

In this study, we aimed to identify tissue-specific genes for various human tissues/organs more robustly and rigorously by extending the tau score algorithm.

**Introduction:**

Tissue-specific genes are a class of genes whose functions and expressions are preferred in one or several tissues restrictedly. Identification of tissue-specific genes is essential for discovering multi-cellular biological processes such as tissue-specific molecular regulations, tissue development, physiology, and the pathogenesis of tissue-associated diseases.

**Materials and Methods:**

Gene expression data derived from five large RNA sequencing (RNA-seq) projects, spanning 96 different human tissues, were retrieved from ArrayExpress and ExpressionAtlas. The first step is categorizing genes using significant filters and tau score as a specificity index. After calculating tau for each gene in all datasets separately, statistical distance from the maximum expression level was estimated using a new meaningful procedure. Specific expression of a gene in one or several tissues was calculated after the integration of tau and statistical distance estimation, which is called as extended tau approach. Obtained tissue-specific genes for 96 different human tissues were functionally annotated, and some comparisons were carried out to show the effectiveness of the extended tau method.

**Results and Discussion:**

Categorization of genes based on expression level and identification of tissue-specific genes for a large number of tissues/organs were executed. Genes were successfully assigned to multiple tissues by generating the extended tau approach as opposed to the original tau score, which can assign tissue specificity to single tissue only.

**Supplementary Information:**

The online version contains supplementary material available at 10.1186/s13040-022-00315-9.

## Introduction

Protein-coding genes in the human genome demonstrate dramatic diversity in terms of expression levels and patterns [1]. Different transcripts are expressed in diverse organs, tissues, or cell types and in different developmental stages. An interesting subset of genes are observed which are strictly expressed in one, or several tissues/organs hence called tissue-specific genes [2, 3]. Identification and analysis of tissue specificity as a dynamic and complex phenomenon, in combination with other biomedical data, provide crucial insights into molecular mechanisms, developmental processes [3, 4], expression-quantitative trait loci [5] and evolution of tissues/organs [6]. Moreover, tissue-specific genes are associated with prognosis, etiology of diseases, and discovery of novel specific drug targets as significant biomarkers for many complex diseases such as solid tumors, neurodegenerative and cardiovascular diseases [7–12]. There have been many studies that examine and determine restricted expression of genes in particular tissues and their relationships with diseases [8, 13–16].

Su et al. [17] and Liang et al. [18] independently generated tissue-specific mRNA expression profiles using a large number of healthy tissue types through microarray. VeryGene tool, which shows relationship between tissue-specific genes with diseases and drugs, was enhanced by Yang et al. [15]. Even though tissue specificity is often used in various researches [19], there is no gold standard method to identify it. Several databases were developed to establish a knowledge base of tissue-specific gene expression in a variety of human tissues. However, consensus among databases is weak due to diverse assumptions, methods, experimental procedures and data types used by those databases [19].

Calculation methods can be divided into two major groups [19] based on their outputs. The first group, including Tau specificity index [20], Gini [19], Tissue Similarity Index (TSI) [21], and Shannon entropy (Hg) [22], produce a single specificity score per gene indicating whether a gene has specific or wide-spread expression. Since genes can be specifically expressed in more than one tissue, producing a single score and pointing out only one tissue is a crucial deficiency of these methods. The second group of specificity calculation methods, which includes Z-score [23], Specificity Measure (SPM) [2], Expression Enrichment (EE) [1], and Preferential Expression Measure (PEM) [24], produce scores as many as the number of tissues for each particular gene and tissue specificity of a gene have to be decided according to threshold values. However, varying thresholds can cause incorrect and inconsistent results.

Previous research has attempted to identify tissue-specific genes with various approaches. Shannon entropy, [22] similar to TSI [19] was used in ROKU, [25] a tool for selection of tissue-specific patterns from microarray data. Tau specificity index was defined as a gene characterization score, and it is a quantitative, graded scalar measurement of specificity of gene expression [20]. Gene Expression and Regulation (TiGER) database [26] was established based on EE score [1], but using obsolete data type and containing data for the low number of tissues renders the database insufficient for extensive research. Z-score [23] approach considers absolute distance from the mean, thus favoring mostly over-expressed genes and occasionally under-expressed genes as tissue-specific genes [19, 27]. In other words, a gene showing housekeeping gene expression with high expression in a tissue would be considered a tissue-specific expression by Z-score calculation. Genotype-Tissue Expression (GTEx) [28] identified tissue-specific genes via Z-score for 53 different tissue types. Both TiSGeD [2] and A Pattern Gene Database (PaGenBase) [29] use SPM to calculate tissue specificity. However, they have a weak correlation in specificity results. PEM calculation proposed by Huminiecki et al. using EST and microarray data from SAGEmap [30], Gene Expression Atlas [31], and TissueInfo [32] databases is a simple form of the EE score [24]. SPM, PEM, and EE are normalized by either maximum expression of a gene or by the sum of gene expressions. Hence they are not sensitive to absolute expression level [19]. Besides,there are some marker gene detection approaches such as CellMapper [33] and Marker Gene Finder in Microarray (MGFM) [34]. However, they are limited to several tissues and/or microarray and EST data omitting RNA-Seq data. Despite presence of multiple methods for calculating tissue-specific expression of a gene, these methods suffer from serious shortcomings. Thus, developing a more robust and rigorous method using more datasets is an important requirement for identifying tissue-specific genes.

Tau is shown to be a more effective method for providing accurate and consistent results in different datasets [19]. It is calculated to determine tissue specificity or sharing of genes across each tissue [35]. However, the tau index is limited to identifying only one tissue in terms of specificity of a gene. In other words, tau can assign a gene to a single tissue, not multiple tissues. Since the definition of tissue-specific genes is considered to be “specifically expressed in one or several tissues”, tau method needs to be improved by additional statistical procedures to assign genes to multiple tissues for specific expression. In this study, estimation of statistically significant interval from maximum expression was calculated to assign a gene to second and/or more tissues for the genes having high tau scores. Therefore, this study makes a major contribution to research on determining tissue-specificity by extending the already effective tau method allowing one-to-many mappings between genes and tissues. Throughout this paper, the term **extended tau** will refer to our novel and rigorous approach for assigning genes to multiple tissues for specific expression. More detailed and accurate tissue specificity of gene expression will enhance understanding evolution of tissues [36–39], relationship between expressions and main functions of genes [20, 40]; and others [41] in various organisms such as mouse [42], *Drosophila* [40] and *Arabidopsis thaliana* [43].

## Methods

### Data retrieval

RNA-seq data for gene expression profiles of 27 human tissues from Fagerberg et. al (EMTAB-1733) [44], 32 human tissues from Uhlen Lab (EMTAB-2836) [45], 53 human tissues from GTEx Project (EMTAB-5214) [28], 56 human tissues from FANTOM5 Project (EMTAB-3358) [46] and 13 human tissues from ENCODE Project (EMTAB-4344) [47, 48] were downloaded via Expression Atlas [49] and ArrayExpress [50]. Detailed information about the raw expression data, number of genes, and tissues are explained in Supplementary Tables 1 and 2, respectively. All calculations were performed using protein-coding genes and tissue types not cell types from the datasets. All tissue types were investigated and grouped according to localization determined via Brenda Tissue Ontology (BTO) [51].

### Categorization of genes based on expression level

Genes were categorized according to their expression level patterns and tau scores. Genes expressed $\le$ 1.0 FPKM or TPM in all tissues were designated as “Null expression” and were excluded from subsequent analysis. Then, expression levels were transformed based on log(2), and the tau score, ranging from 0 to 1, was calculated for each gene [19] using the formula below where $x_{i}$ is expression of a gene in tissue *i* and *n* is number of tissues.

$$\begin{aligned} \tau =\frac{ \sum _{i= 1}^{n}\left( 1-\hat{x}_{i} \right) }{n-1} \end{aligned}$$

$$\begin{aligned} \hat{x}_{i}= \frac{x_{i}}{\max _{1 \le i\le n} x_{i}} \end{aligned}$$

If tau score is $(\tau )\ge 0.85$ for a given gene, that gene is marked to have *Specific expression*. The genes having $\tau < 0.85$ were classified as *Wide-spread expression*. Genes which have expression values $< 10$ in all tissues was denoted as *Weak expression* [8]. Tau score was calculated for weakly expressed genes but their scores were ignored during tissue specificity assessment. Log transformation was used only during tau calculation; after that, all other calculations were performed using raw expression values. Rigorous tissue-specificity classification was proceeded with the genes with $\tau \ge 0.85$ and expression value $> 10$ in all tissues.

### Estimation of statistically significant interval

F-test [52] was used to verify the equality of variance between datasets. Statistically significant distance from the maximum expression value was calculated in order to assign genes to multiple tissues in the context of specificity. For this purpose, the lower and upper bounds of raw expression data were calculated via Fuzzy c-means clustering [53]. Ratio of upper cluster was calculated for each tissue with the following formula where $n_{up}$ is the number of elements in upper cluster and $n_{total}$ is the number of total non-zero elements.

$$\begin{aligned} ratio= \frac{n_{up}}{n_{total}} \end{aligned}$$

Regression analysis [54] was used to calculate an optimized threshold value of *ratio* and then converted it to Z-value using the inverse function of normal distribution for each dataset. Assessment of normality of datasets was performed by Kolmogorov-Smirnov (K-S) test [55] and Q-Q plots. The equation below was used to calculate statistically significant distance $(dist_{ss})$ where $x_{max}$ is the maximum expression value of a gene among all tissues, $\sigma$ is the standard deviation of non-zero expression of a gene among all tissues and $z_{val}$ is optimized threshold as Z-value.

$$\begin{aligned} dist_{ss}={x_{max}-\sigma \times z_{val}} \end{aligned}$$

**Fig. 1:**
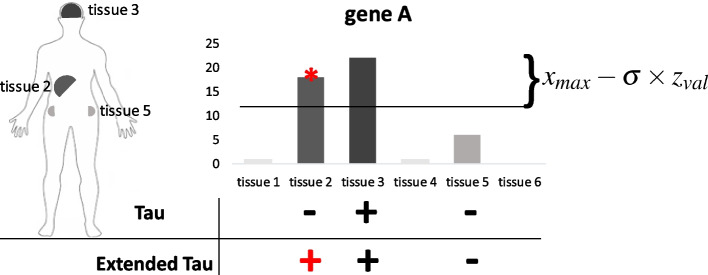
Illustration of extended tau approach. Gene can specifically expressed one or more different tissues. Here, gene A is specifically expressed in tissue 2 and tissue 3. Extended tau can determine both of two compared to only tau calculation

All calculations were performed for all datasets separately, and threshold ratios are available in Supplementary Table 3. Integration of tau score with the statistically significant interval from maximum expression was described as **extended tau** for robust and rigorous identification of tissue-specific genes via assignment of genes to possible multiple tissues. Extended tau approach is illustrated in Fig. 1.

### Functional annotation of tissue-specific genes

Database for Annotation, Visualization and Integrated Discovery (DAVID) [56] was used to identify the roles of specific genes in biological processes, potential functions, related tissues, and diseases. Results were compared to GeneCards [57] which already integrated expression data from GTEx [28], Illumina Body Map [58], BioGPS [59], and CGAP SAGE [60]. Moreover, The Human Protein Atlas [45] was also used to compare transcript levels of tissue-specific genes with their protein levels.

## Results and Discussion

Tau score is a robust method to identify tissue-specific genes [19] but is limited in its capacity to match genes with multiple tissues. To overcome this, we developed a new extensive procedure where the specific expression of a gene in one or several tissues was calculated by integrating tau score with statistical distance as a new rigorous approach described as extended tau calculation.

RNA-Seq data from five studies aimed to determine gene expression in multiple tissues were retrieved from publicly available databases. When all datasets are combined, expressions from the total of 96 different tissues are represented. The tissues had parent-child relationships with various depths in the hierarchy. After assigning parent-child mappings using Brenda Tissue Ontology (BTO), 96 different tissue types, referred to as *child tissue*, were mapped to 36 top-level tissues, referred to as *parent tissue*. All tissue types and their BTO accession IDs are listed in Supplementary Table 4.

**Fig. 2:**
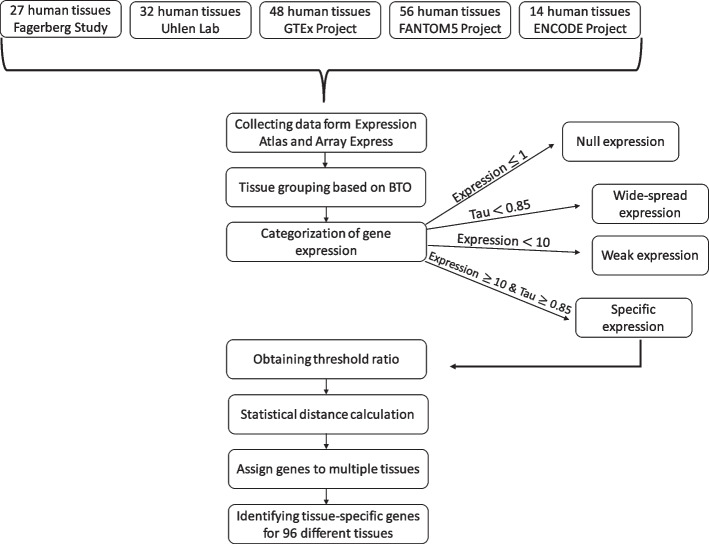
Workflow for the identification of tissue-specific genes. Firstly, genes were categorized based on some significant filters and tau calculation. After that, statistical distance estimation was used to determine specific genes in a rigorous manner

A summary of the workflow for categorizing protein-coding genes and calculating tissue-specific genes using extended tau is presented in Fig. 2, and a detailed workflow is shown in Supplementary Fig. 1. F-test was used to demonstrate whether there was a significant difference among datasets. According to F-test results, expression values in datasets were found to have equal variances (Supplementary Table 5). The higher F-score value for the dataset EMTAB-3358 is due to the fact that the dataset has units of TPM compared to FPKM in other datasets. Therefore, EMTAB-3358 is distinct from other datasets; still, there is no significant difference among the datasets. Boxplots and violin plots showing the distribution of expression levels for each dataset are shown in Supplementary Fig. 2. Kolmogorov-Smirnov test shows that datasets have a normal distribution, and Q-Q plots for each dataset are presented in Supplementary Fig. 3.

According to criteria described in Fig. 2, the genes were categorized based on their expression level profiles and tau scores. Table 1 summarizes number of genes in each category for each dataset. Please note that total number of genes in the Table 1 matches the number of filtered genes in Supplementary Table 2.

**Table 1:** Number of genes in each category for all datasets

Gene profile	Fagerberg Study	Uhlen Lab	GTEx Project	FANTOM5 Project	ENCODE Project
Null expression	1,260	2,427	2,672	1,808	3,394
Weak expression	1,808	2,533	2,788	3,869	2,976
Wide-spread expression	13,126	11,733	11,434	8,698	11,013
Specific expression	2,669	2,983	2,782	2,063	2,293
Total number of genes	18,863	19,676	19,676	16,438	19,676

According to Table 1, number of genes showing specific expression is comparable among all samples. After this categorization, statistically significant interval from maximum expression was applied to genes showing specific expression to assign them to multiple tissues. As expected, the extended tau approach reveals more gene-tissue pairs when compared to original the tau calculation as listed in Table 2.

**Table 2:** Number of specific gene-tissue pairs based on tau and extended tau calculations

Datasets	Tau	Extended Tau
Fagerberg Study	2,669	3,370
Uhlen Lab	2,983	4,257
GTEx Project	2,782	4,680
FANTOM5 Project	2,063	3,982
ENCODE Project	2,293	3,097

**Fig. 3:**
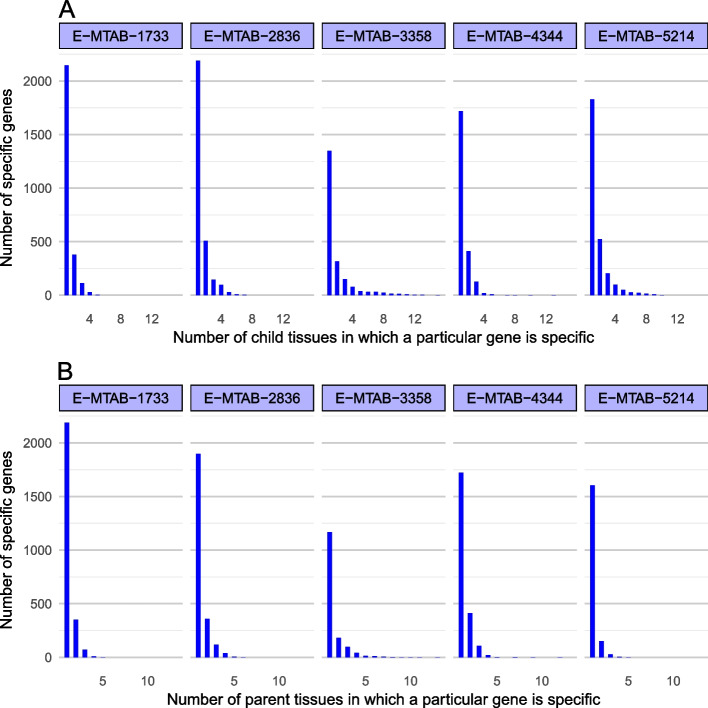
Distribution of tissue-specific genes. Extensive graph shows how many genes are specific in how many tissues per dataset for all child (A) and parent tissues (B). In each plot, the leftmost bar represents number of tissue-specific genes that are specific to single tissue. Remaining bars show number of genes that are specific to multiple tissues which was calculated by multiple assignment of genes to tissues based on extensive tau

Although the tau scores of genes and the number of tissue-specific genes are same between two approaches, the extended tau approach provides more gene-tissue mappings for specific expression. The extended tau approach has ability to list gene-tissue mappings for genes specific to one or several tissues [2, 3]. The number of specific genes per tissue is provided in Supplementary Table 6, and Fig. 3 summarizes number of tissue-specific genes distributed by number of gene-tissue mappings for both parent and child tissues in each dataset. Majority of genes were expressed specifically to only one single tissue and some genes are specifically expressed in two or more different tissues regardless of tissue hierarchy, parent or child.

We used DAVID and other resources to show coherences between our results and the functions of several genes. Alpha fetoprotein (AFP) is a liver-specific gene [61]; however, it is defined not only liver-specific but also kidney-specific gene based on extended tau. One of its related pathways is the glucocorticoid receptor regulatory network, and the level of AFP in amniotic fluid is used to measure renal loss of protein. Intestinal Alkaline Phosphatase (ALPI), which encodes a digestive brush-border enzyme, has a specific expression in the small intestine based on the tau calculation, although extended tau demonstrates that ALPI is specifically expressed in both small intestine and duodenum. Another example is MAP7 Domain Containing 2 (MAP7D2) which contributes to the structural integrity of a complex is specifically expressed in the brain based on tau, even though it is also specifically expressed in the testis. D-amino acid oxidase (DAO) has specific expression in kidney according to tau score. On the other hand, it was noticed that DAO is specific to brain and liver after calculation of extended tau. DAO may act as a detoxifying agent which removes D-amino acids that accumulate during aging. It is generally related to some neurological diseases. Shortly, extended tau is a more comprehensive approach to find several tissues for one specific gene.

The brain is a complex organ characterized by a high level of gene expression; at least 30-50% of approximately all protein-coding genes are expressed across all parts of the brain and it has a significant variety of functions [62, 63]. Significant differences in cell composition of the various anatomical brain regions result in cell-specific differences in gene expression. There are many specific genes all over the brain as parent tissue [64]. Comparison in terms of tissue-specific genes in child tissues of brain was not performed because, there are many different brain parts coming from different datasets. It can be stated that brain-specific genes are more often selectively expressed in either neurons or glial cells and vascular cells from the cerebral cortex [65]. The cerebral cortex has a higher number of specific genes shown in Supplementary Fig. 4 as child tissue.

The datasets used in this study did not examine the same set of tissues. Therefore, a gene is not necessarily specific in a particular tissue, according to five datasets. If five datasets have a certain consensus presented in Supplementary Fig. 5 for the specificity of a particular gene, we can be sure that it is absolutely specific to related tissue. For instance, 252 genes are specific to the testis according to all five datasets, and 262 genes are specific to the testis supported by 4 datasets shown in Supplementary Fig. 5. Agreement of datasets is important for accuracy. On the other hand, if tissues are included in only one single dataset, genes specific to that tissue will be supported by only one dataset, naturally. Gene expression in whole blood, tongue, epididymis, seminal vesicle, tibial nerve, Vas deferens, and spinal cord are examined in only one dataset. Therefore, genes specific to these tissues are supported by one dataset.

**Fig. 4:**
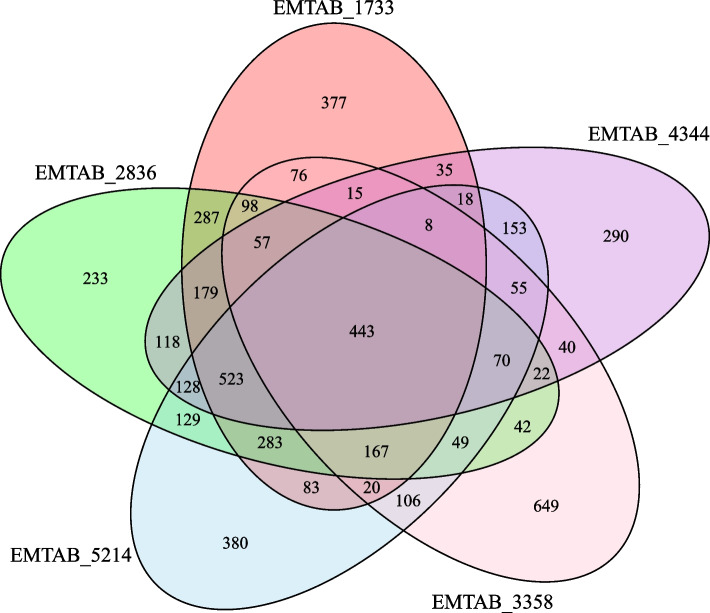
Venn diagram of tissue-specific gene lists derived from five different datasets. Comparison of results depends on the number of tissue-specific genes was illustrated via Venn Diagram. Intersected genes among five datasets are absolutely specific to related tissues

Figure 4 summarizes the comparison of tissue-specific gene lists across different datasets. Four hundred eighty-four genes were found to be tissue-specific according to all datasets. Although some genes are found to be specifically expressed in only one dataset, this result suggested that our approach is suitable and effective for determining tissue-specific genes. Comparison is very important for the correctness and reliability of the results. Naturally, there are differences in the results as they are created with different experimental and laboratory conditions, samples, and also different normalization methods during obtaining RNA-seq data. If we examine Fig. 4, 692 genes are determined to be tissue-specific by only EMTAB-3358 (FANTOM5 Project) because expression values were normalized using a different method, despite the same experimental procedure.

The correlation of raw expressions of datasets is demonstrated in Supplementary Fig. 6, and similarity is defined as a dark color; the size of nodes shows the number of genes common between two datasets. It can be shown that the similarity of EMTAB-5214 (GTEx Project) and EMTAB-2836 (Uhlen Lab) is higher than any other dataset pairs, according to Supplementary Fig. 6. EMTAB-3358 (FANTOM5 Project) is quite different from all other datasets, as observed in the previous results. After examining the datasets for similarity of raw gene expression, the datasets were compared to each other to understand the correlation, reliability, and effectiveness of the extended tau method. Genes have a tau score greater than 0.85, and an accompanying correlation plot has been drawn in Supplementary Fig. 7. It was shown that EMTAB-4344, EMTAB-1733, and EMTAB-2836 give similar results after determining tissue specificity. As before, EMTAB-3358 is also far from the other datasets. Two data that provide the closer results are EMTAB-1733 and EMTAB-2836. On the other hand, the two data that give the most distant results are EMTAB-3358 and EMTAB-4344, according to Supplementary Fig. 7.

Tissue-specific expression profiles can be used for biomedical applications such as tissue-specific regulation [66] of genes, examining gene profiles for various disorders, enhancing the efficiency of therapies, discovering new biomarkers for diagnosis and also targeted treatment of diseases such as cancer [67] and malignancies [68]. Organogenesis is another important phenomenon related to biological processes [36, 37]. The progression of tissues, organs, and systems in living organisms can be understood by identifying tissue-specific genes and their roles. Besides, interpretation of relationship between tissues in the context of specific genes is a very crucial approach to find out not only tissue progression but also the discovery of mechanisms of diseases. Common tissue-specific genes between different tissues might give clues to unravel relationships between various tissues. Interestingly, the brain has connections to all of the tissues because brain expression may reflect developmental ontogeny, or developmental stages processes of the human body [62, 69].

Bone marrow, spleen, and lymph node are tightly connected in terms of specific genes and it is known that they are members of the lymph system [70]. A group of tissues is related to female reproductive system, including vagina, uterus, ovary, and oviduct [71] that express a list of common specific genes. Another case concerns human digestive system organs which are small intestine, colon, rectum, liver, and stomach which have some common tissue-specific genes. However, it has also been observed that some specific genes related to digestive system are associated with kidney. Different organs/tissues can have similar subsequent processes such as ammonia-urea conversion [72, 73] and the single gene can be specific to several organs. In addition, epididymis, Vas deferens, penis, and testis are tightly connected to each other as parts of male reproductive system. They have shared tissue-specific genes according to the extended tau results and our findings are consistent with both literature and Brenda Tissue Ontology (BTO).

Although all tissues carry out common processes in the human body, tissues can be distinguished by gene expression levels [74]. After filtering out transcripts with low-level and wide-spread expressions, protein-coding genes were assessed for specific expression in tissues using a robust and rigorous calculation, extended tau. Tissue-specific genes were successfully assigned to multiple tissues and identified with great care.

## Conclusion

This study provides a large insight into tissue specificity. Tissue-specific genes for 96 different tissue samples from the human body via five RNA-seq datasets were calculated by the extended tau approach. Tau score is a more effective and accurate calculation method, and the statistical distance term is meaningful for assigning genes to several specific tissues. After the categorization of protein-coding genes and identification of tissue-specific genes in a broad sense, their functional properties were investigated. It can be suggested that tissue specificity results will benefit further studies to reveal molecular mechanisms of healthy tissues and diseases.

The extended tau approach can be used in other regulatory elements like transcription factor (TF) [74]. TFs may have higher tissue specificity, because tissue-specific processes are ultimately controlled by gene regulatory networks [74, 75]. Therefore computational analysis of tissue-specific TFs and other regulatory networks will provide a critical perspective.

RNA-Seq data of a single tissue can include genes which are specific to other tissues after calculation of tissue specificity. This condition may be related to change of expression level or migration of cells which are originated from other tissues/organs. When tissue heterogeneity, cell migration, change of expression level or behaviors of genes would like to be examined, the tau score will be incomplete. In this situation, the extended tau approach can give more robust and rigorous results for various research.

## Supplementary information

Additional file 1. Algorithm to determine tissue specific genes.

Additional file 2. Distribution of gene expression levels in all datasets - violin and boxplots.

Additional file 3. Distribution of gene expression levels in all datasets.

Additional file 4. Number of genes for child and parent tissues.

Additional file 5. Number of genes per tissue in all the datasets.

Additional file 6. Correlation of raw expressions of datasets.

Additional file 7. Correlation of gene expressions for genes which have tau score greater than 0.85.

Additional file 8. Descriptive summary of all datasets.

Additional file 9. Number of genes and tissues in each dataset.

Additional file 10. Optimized thresholds and Z-scores for each dataset.

Additional file 11. Determining parent-child tissue relationship.

Additional file 12. F-test results for each pair of datasets.

Additional file 13. Number of tissue specific genes for each tissue.

## Data Availability

Codes are available at https://gitlab.com/busra/modified_tau.

## Abbreviations

EE: Expression Enrichment
FPKM: Fragments per kilobase per million reads
GTEx: Genotype-Tissue Expression
Hg: Shannon entropy
PaGenBase: Pattern Gene Database
PEM: Preferential Expression Measure
RNA-Seq: RNA sequencing
SPM: Specificity Measure
TiGER: Gene Expression and Regulation
TPM: transcript per million
TSI: Tissue Similarity Index
