# Supplementary figures and images for: Robust and rigorous identification of tissue-specific genes by statistically extending tau score
Source: BioData Min. 2022 Dec 9;15:31. doi: 10.1186/s13040-022-00315-9 (PMC9733102; doi:10.1186/s13040-022-00315-9)

# Determination of Tissue Specific Genes - Algorithm

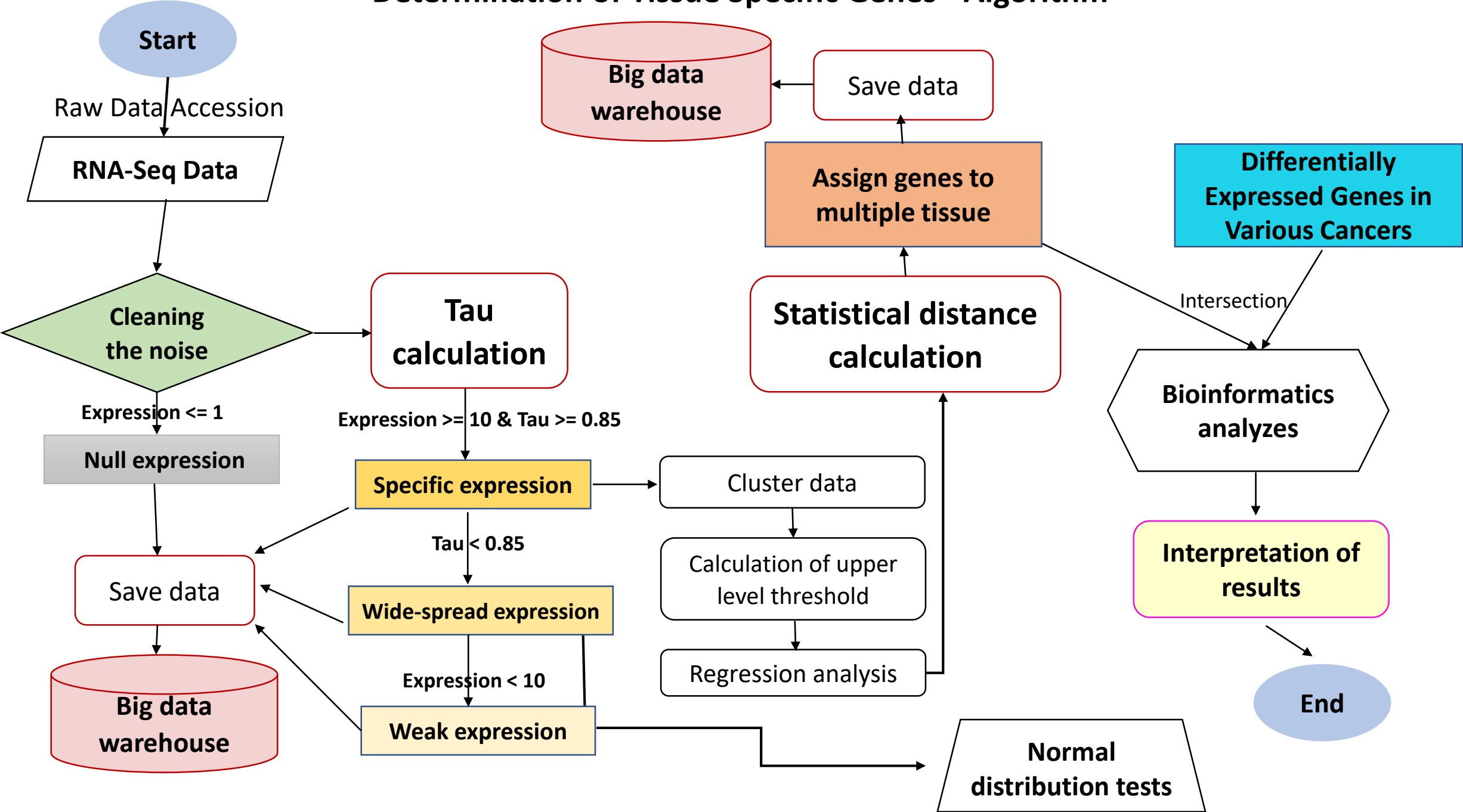

Supplement: Supplementary file 1 — Additional file 1. Algorithm to determine tissue specific genes. [file 13040_2022_315_MOESM1_ESM.pdf]

**A**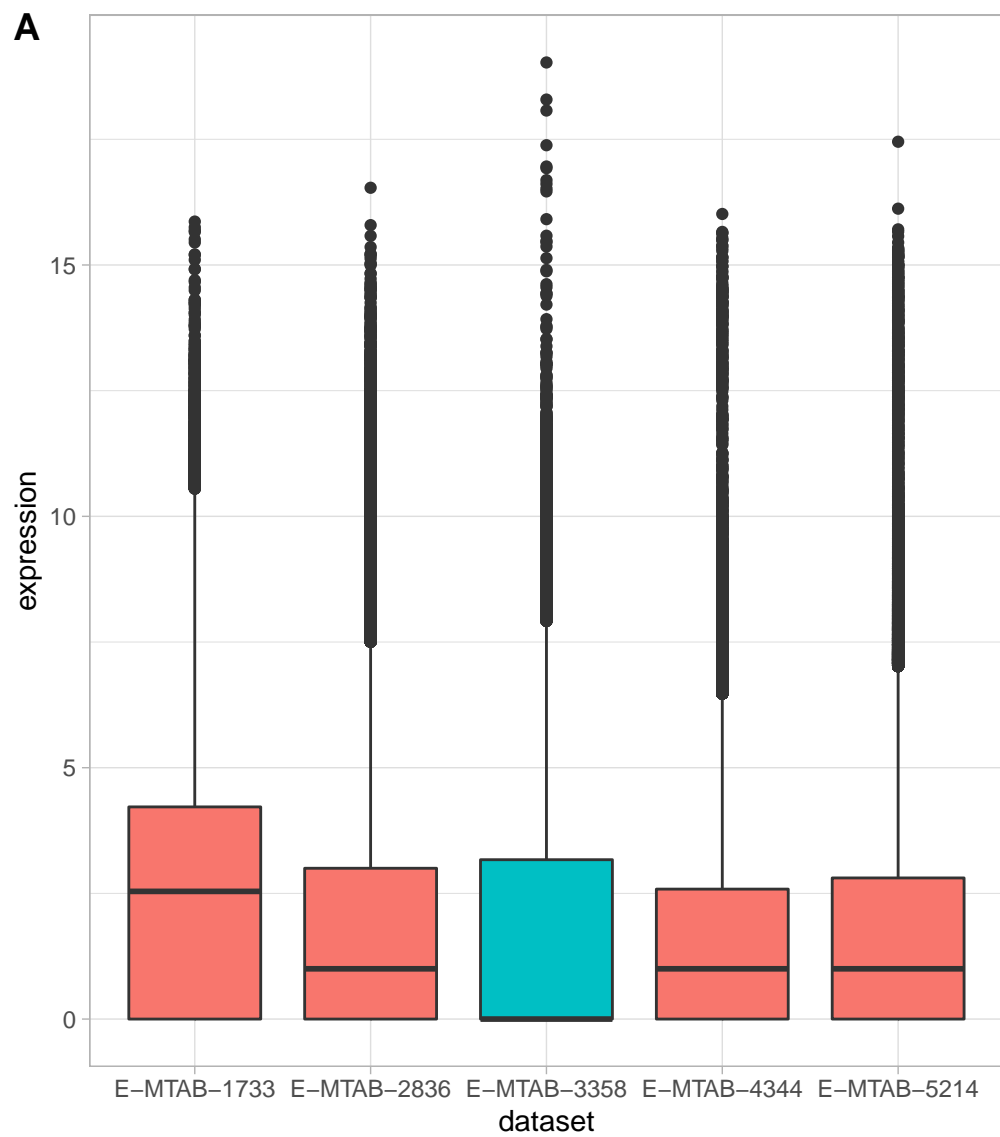**B**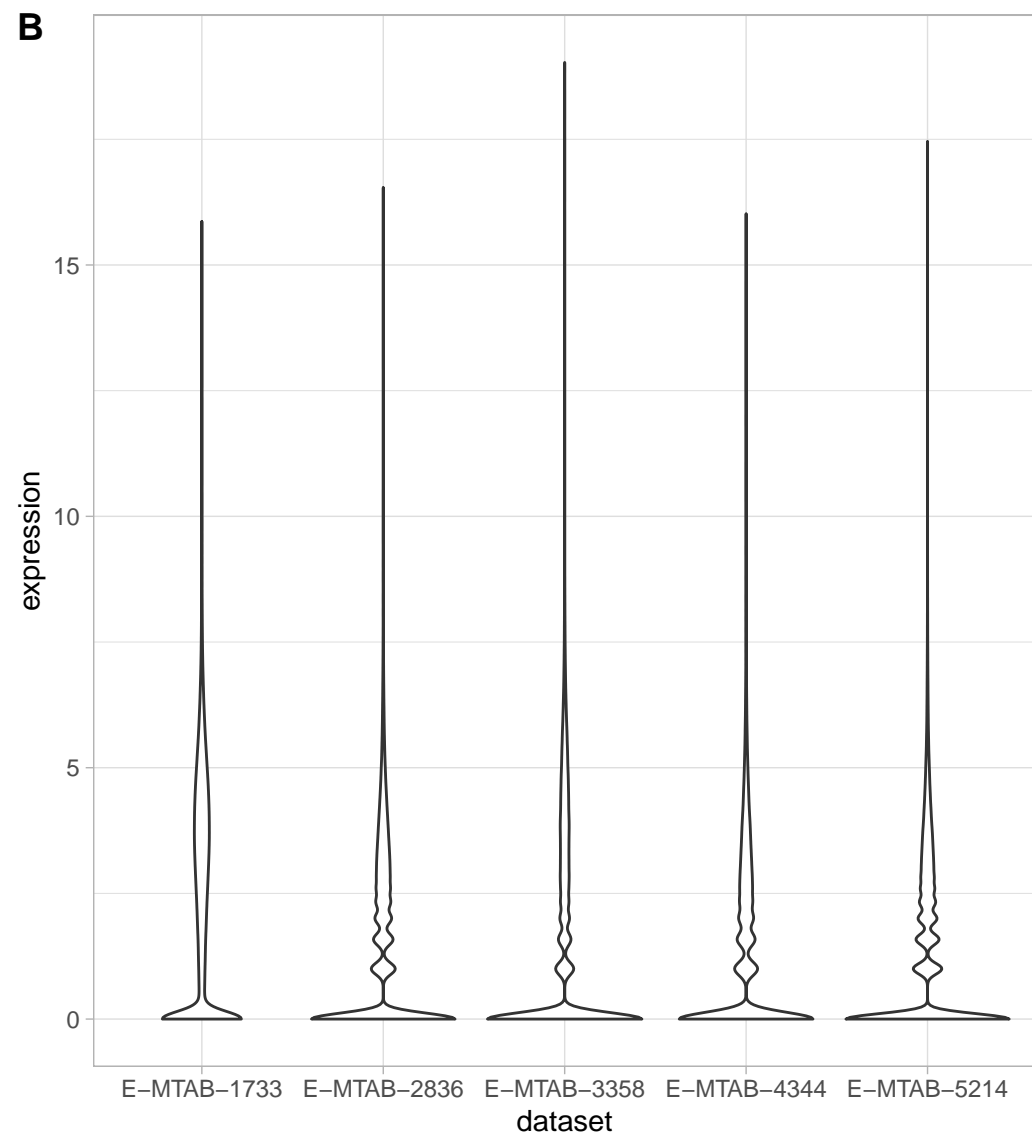

Supplement: Supplementary file 2 — Additional file 2. Distribution of gene expression levels in all datasets - violin and boxplots. [file 13040_2022_315_MOESM2_ESM.pdf]

Child tissues

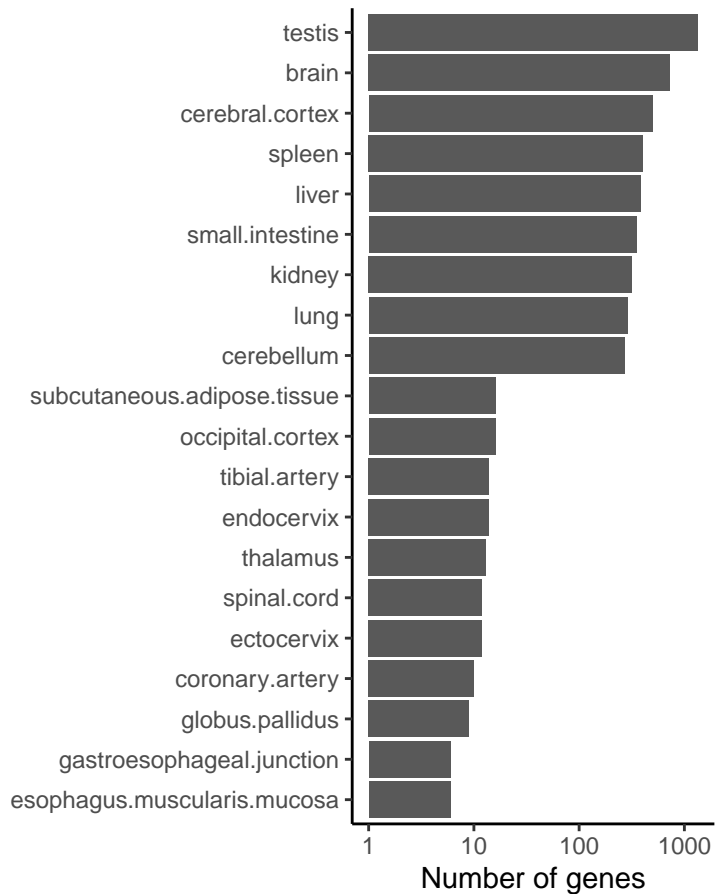

Parent tissues

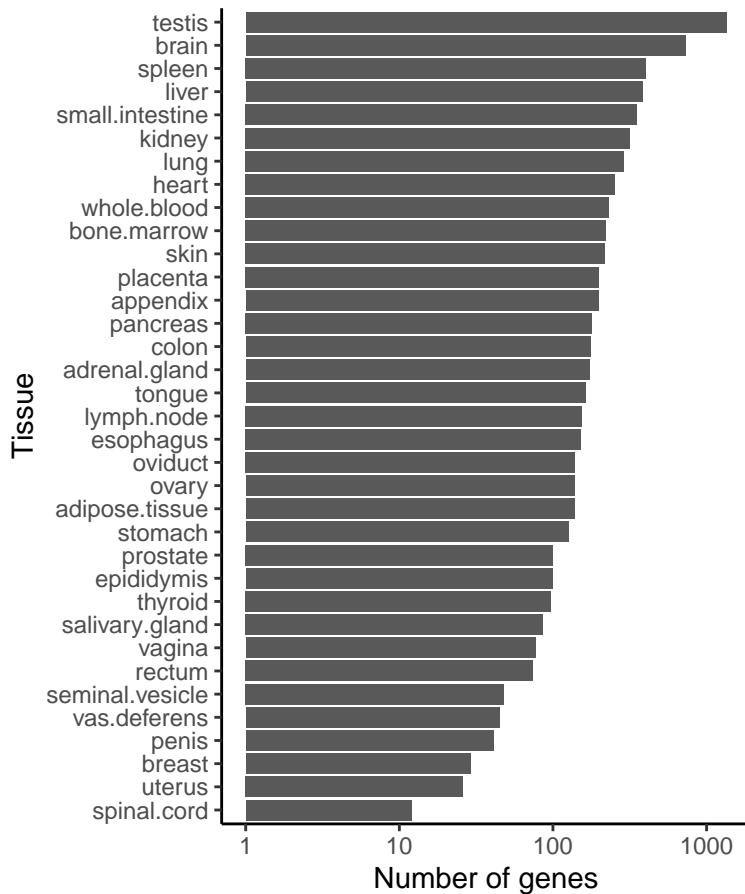

Supplement: Supplementary file 4 — Additional file 4. Number of genes for child and parent tissues. [file 13040_2022_315_MOESM4_ESM.pdf]

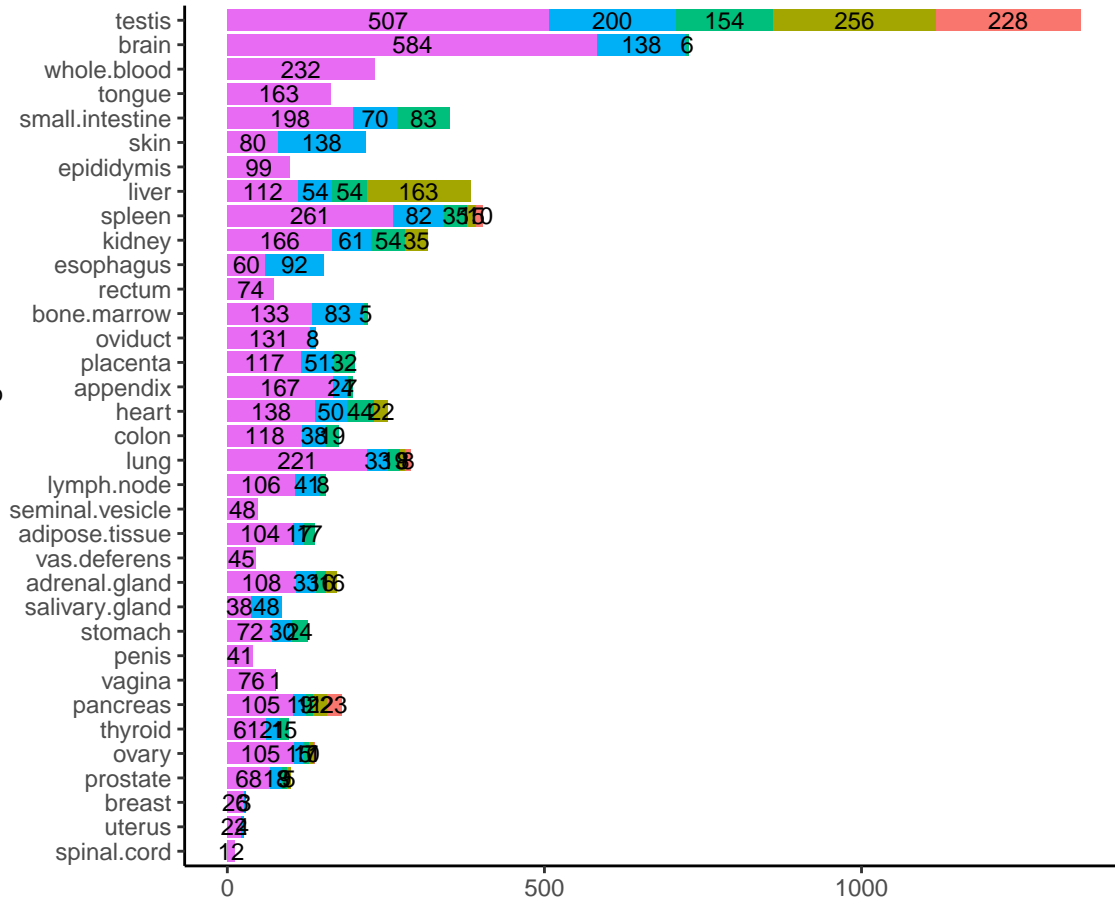

## Agreement of Datasets

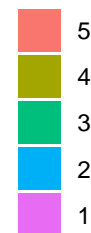

Supplement: Supplementary file 5 — Additional file 5. Number of genes per tissue in all the datasets. [file 13040_2022_315_MOESM5_ESM.pdf]

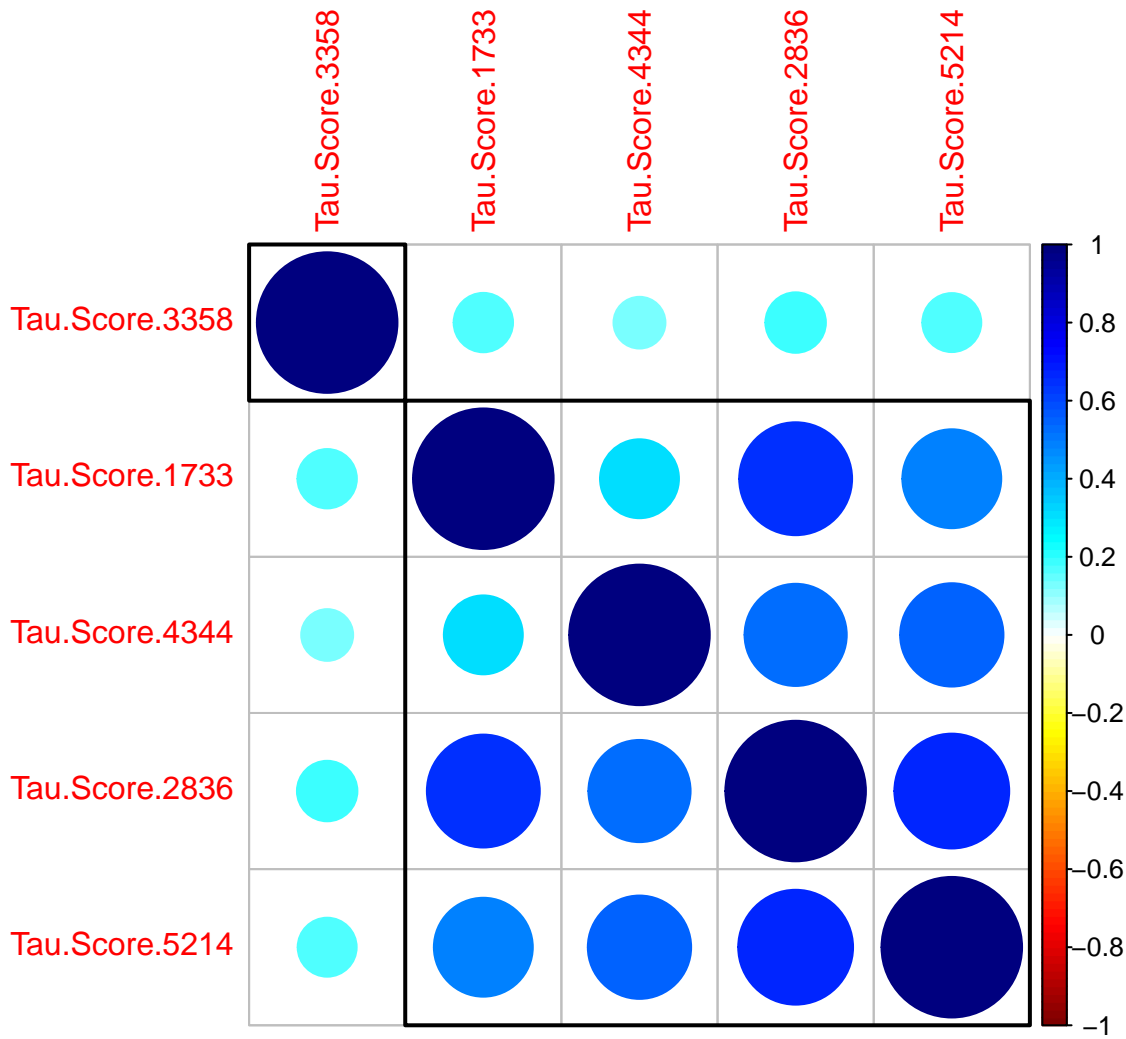

Supplement: Supplementary file 6 — Additional file 6. Correlation of raw expressions of datasets. [file 13040_2022_315_MOESM6_ESM.pdf]

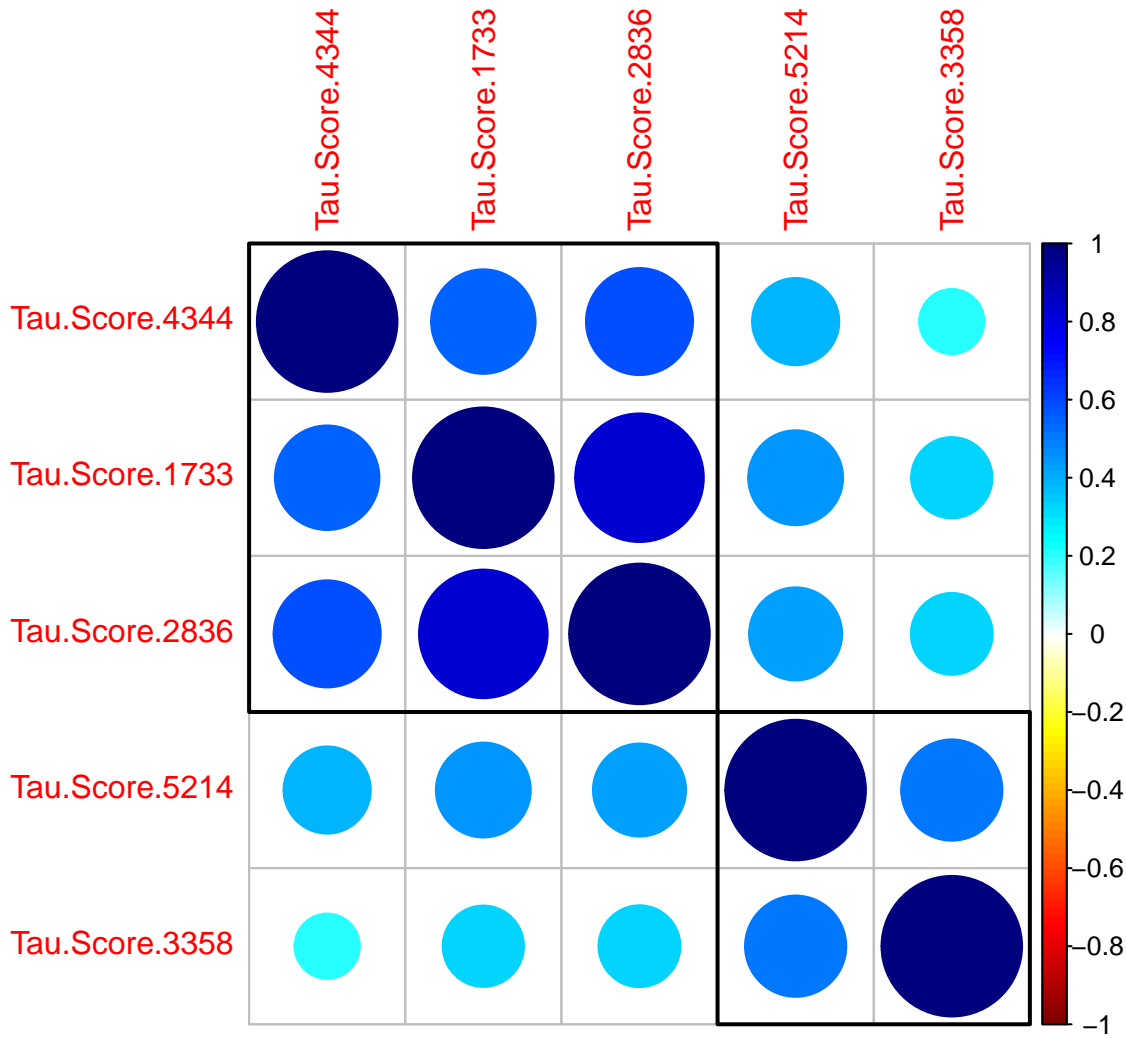

Supplement: Supplementary file 7 — Additional file 7. Correlation of gene expressions for genes which have tau score greater than 0.85. [file 13040_2022_315_MOESM7_ESM.pdf]
