# Supplementary material for: Robust and rigorous identification of tissue-specific genes by statistically extending tau score
Source: BioData Min. 2022 Dec 9;15:31. doi: 10.1186/s13040-022-00315-9 (PMC9733102; doi:10.1186/s13040-022-00315-9)
Supplement: Supplementary file 3 — Additional file 3. Distribution of gene expression levels in all datasets. [file 13040_2022_315_MOESM3_ESM.pdf]

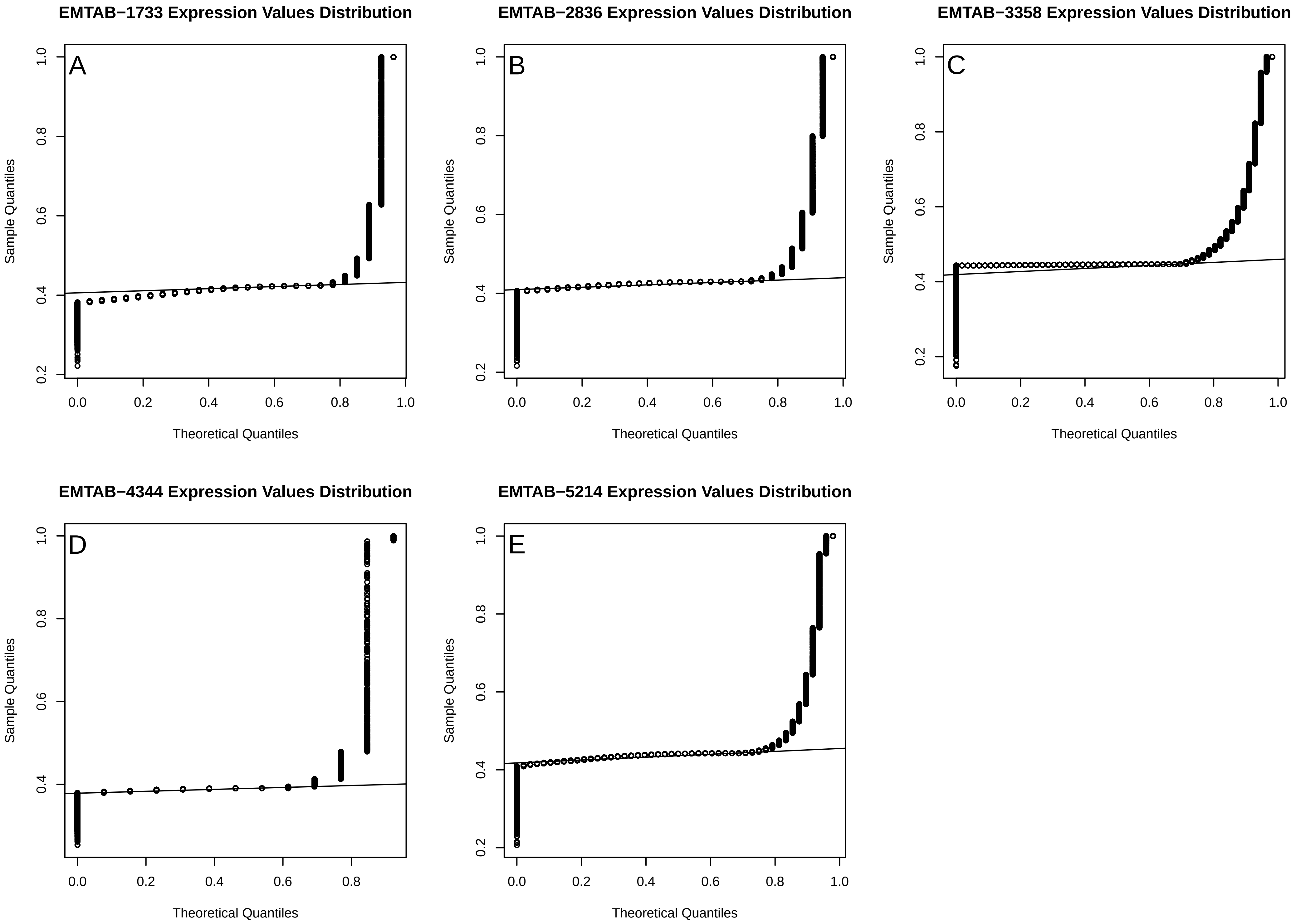

EMTAB−1733 Expression Values Distribution
A
EMTAB−2836 Expression Values Distribution
B
EMTAB−3358 Expression Values Distribution
C
EMTAB−4344 Expression Values Distribution
D
EMTAB−5214 Expression Values Distribution
E
Sample Quantiles
Theoretical Quantiles
0.0
0.2
0.4
0.6
0.8
1.0
